# The Pharmaceutical Society and the Sale of Laudanum

**Published:** 1916-02-26

**Authors:** 


### The Hospital

The Workers' Newspaper of Administrative Medicine and Institutional Life, Administration, National Insurance and Health.

No. 1550, Vol. LIX. SATURDAY, FEBRUARY 26, 1916. PRICE ONE PENNY.

### THE PHARMACEUTICAL SOCIETY AND THE SALE OF LAUDANUM.

In our issue of September 11, 1915, we drew the attention of our readers to a somewhat unfortunate controversy which had arisen relative to the sale of "laudanum," and it is now with much satisfaction that we are able to record the closing of this controversy in a fashion creditable to all concerned. The difficulty, it may be remembered, was a consequence of the issue, somewhat more than a year ago, of the new edition of the British Pharmacopœia. In this issue "tincture of opium" and "laudanum" were made synonymous terms, and the strength of the preparation was increased to such a level that it contained 1 per cent. of morphine. As a result of the increased strength the tincture came automatically under the operation of the provisions defined in Part I. of the Schedule to the Pharmacy Act—that is, any sale of it required entry in a "Poisons Book" and could only be effected if the purchaser was either personally known to the pharmacist or was introduced by some person known to them both. According to the earlier Pharmacopœia, tincture of opium or laudanum had only had a strength of 0.75 per cent. of morphine. Hence it fell into Part II. of the Poisons Schedule and its sale took place across the counter in the ordinary fashion and without the necessity for any formal record. The alteration in the strength of the preparation thus altered its legal position, and not unnaturally a public which had been accustomed to purchase "laudanum" as a mere commercial transaction was disturbed by the new conditions and was disposed to resent them; and, again, customers who had long been accustomed to a preparation of a certain strength were reluctant to allow that a stronger tincture should be supplied to them. The public, in short, wished to keep to the older preparation and to the easier conditions.

This desire of the public placed the pharmacist in a very embarrassing position. To explain to a freedom-loving Briton that the laudanum to which he has become attached has been increased in strength and that the purchase of even the smallest quantity demands an elaborate ceremony is not an easy task, and the process not infrequently did, as a matter of fact, excite friction. This may seem a very trivial affair to persons who neither purchase laudanum nor sell it. But to pharmacists and their customers the position created a substantial grievance, for the purchase of laudanum in small quantities as a domestic remedy for various minor ailments is quite a common practice. Doubtless there are forcible persons who will say that such a practice ought to be made illegal. Perhaps so; but the fact remains that it is not illegal, and there is no duty incumbent on the pharmacist to constitute himself the censor of a custom which the law allows and which finds favour with a considerable section of public opinion. His duty is to obey the law, and this he does when he supplies his customers with laudanum under the conditions prescribed by Act of Parliament. The difficulty of his situation arose from the fact that the sale of "laudanum" by the action of the General Medical Council had been brought under restrictions which did not previously apply to it. In these circumstances it was hardly surprising to find that some pharmacists, having explained the position to the purchaser, continued to supply "laudanum" of the old standard, and thus avoided the irksome conditions of a record in the Poisons Book. What the exact legal quality of such an action may be is a matter for the lawyers, but as a matter of general principle it seems difficult to deny the right of a pharmacist to sell to his customer the articles this latter desires. So long as the transaction is perfectly open and frank there appears nothing morally objectionable in supplying or in purchasing a preparation made according to the standard, not of the current, but of a previous Pharmacopœia. Certainly such a freedom is open to the physician in writing his prescription, and, if so, why not to the private purchaser? The question came inevitably to the attention of the Pharmaceutical Society, which authoritatively issued a formula wherein the right just mentioned was claimed, recognising at the same time the duty of supplying the new standard unless the older and less powerful preparation was demanded. In course of time reference was made to the General Medical Council, and there seemed at one time every prospect of an acute controversy which might well have reached a final settlement only through the agency of the courts of law. Happily, a reasonable and even a generous spirit has prevailed, and the Pharmaceutical Society has taken a step which in substance will avoid the unfortunate position that would exist were two preparations differing in strength the one from the other to be known by one and the same name.

What the Society has done is to agree that Part I. of the Poisons Schedule shall now apply to preparations which contain 0.75 per cent. of morphine, and this brings the "old" laudanum equally with the "new" into a position which demands the entry of every sale in the Poisons Book and its attestation by the signature of the purchaser. Hence the trouble to purchaser and to salesman is the same in the two cases, and there is no temptation to maintain the two standards, seeing that the same legal formalities apply in both instances. Though we do not agree with those who urge that no active drug should be supplied except on the order of a physician's prescription, we cordially support the measures which warn the public that in purchasing such agents there are risks and dangers. Hence we are quite content that the sale of laudanum shall be conditioned by the formalities above described. They announce to the public that poisonous drugs are edged tools which require to be used with discretion. If we may venture to do so, we will congratulate the Pharmaceutical Society on its decision. For individual pharmacists it means a good deal of trouble, but these must try to solace themselves with the conviction that in the ungrateful task of educating the public they are acting in harmony with the best traditions of their craft.

